# Pathological and protective roles of dendritic cells in *Mycobacterium tuberculosis* infection: Interaction between host immune responses and pathogen evasion

**DOI:** 10.3389/fcimb.2022.891878

**Published:** 2022-07-28

**Authors:** Hongmin Kim, Sung Jae Shin

**Affiliations:** Department of Microbiology, Institute for Immunology and Immunological Diseases, Graduate School of Medical Science, Brain Korea 21 Project, Yonsei University College of Medicine, Seoul, South Korea

**Keywords:** *Mycobacterium tuberculosis*, dendritic cells, pathogenesis, protective immunity, vaccine, host-directed strategy

## Abstract

Dendritic cells (DCs) are principal defense components that play multifactorial roles in translating innate immune responses to adaptive immunity in *Mycobacterium tuberculosis* (Mtb) infections. The heterogeneous nature of DC subsets follows their altered functions by interacting with other immune cells, Mtb, and its products, enhancing host defense mechanisms or facilitating pathogen evasion. Thus, a better understanding of the immune responses initiated, promoted, and amplified or inhibited by DCs in Mtb infection is an essential step in developing anti-tuberculosis (TB) control measures, such as host-directed adjunctive therapy and anti-TB vaccines. This review summarizes the recent advances in salient DC subsets, including their phenotypic classification, cytokine profiles, functional alterations according to disease stages and environments, and consequent TB outcomes. A comprehensive overview of the role of DCs from various perspectives enables a deeper understanding of TB pathogenesis and could be useful in developing DC-based vaccines and immunotherapies.

## Introduction

Tuberculosis (TB), caused by the *Mycobacterium tuberculosis* (Mtb) complex, is one of the most successful infectious diseases in humans, resulting in millions of TB cases annually. The global estimate of TB cases in 2020 was 10 million (WHO Global Tuberculosis Report 2021). Although recent advances in the development of new vaccines and immunomodulatory drugs would provide a more effective means of fighting Mtb infection, the precise mechanisms of protective and pathological immunity have not been fully elucidated.

TB is commonly initiated by the inhalation of respiratory droplet nuclei (≤1–2 mm) containing Mtb, which are small enough to pass down the respiratory tract and into the alveoli (Schluger and Rom, 1998). Cell types, such as macrophages, neutrophils, dendritic cells (DCs), and permissive monocytes, can be infected by Mtb. However, the alveolar macrophages are primarily the initially infected cells from which Mtb is disseminated to the lung interstitium (Cohen et al., 2018). The relocalization of alveolar macrophages enables recruited macrophages, neutrophils, and DCs to phagocytose Mtb, resulting in the formation of initial granulomas (Cohen et al., 2018). Subsequently, along with other cells, the Mtb antigen (Ag)-specific T cells infiltrate the infection site, progressing granuloma formation to control Mtb.

DCs are major cell populations capable of presenting Mtb-specific Ags to T cells using major histocompatibility complex (MHC) class I and class II molecules along with various cytokines. Proinflammatory cytokines, such as the IL-1 family and IL-6, promote the recruitment of immune cells (Giacomini et al., 2001) for effective defense. Importantly, mature DCs following Mtb infection or Ag-uptake migrate to the draining lymph nodes (dLNs) and promote pathogen recognition by T cells, resulting in specific T cell polarization in the diverse microenvironments of infection sites (Marino et al., 2004). Distinct populations of CD4^+^ T helper (Th) cells differ based on cytokine profiles, transcription factors, and their responses to various classes of pathogens. The immune response against intracellular bacteria, such as Mtb and Ag-specific IFN-γ-producing Th1, is a key factor in restraining Mtb growth (Flynn et al., 1993). After the adaptive immune response is initiated, DCs continuously uptake Mtb-Ags to induce a systemic T cell response while moving in and out of granulomas following granuloma formation and are continuously replaced during Ag sampling (Harding et al., 2011; Schreiber et al., 2011a). They can also regulate local T cell responses and can carry bacteria into the lymph nodes, which is crucial for generating systemic T cell responses (Harding et al., 2015).

The induction of these T cell-related protective immune responses has been studied focusing primarily on Ag acquisition by DCs at the infection site in the early stage of infection and the interaction with T cells in the LNs. However, DCs can be affected by their interactions with various cell types and immunological environments. Thus, a better understanding is important for TB control by developing improved vaccines and control strategies based on additional research into the DC cell population, including host-directed therapy (HDT). In this review article, we address the molecular and cellular aspects of DCs according to their subsets and interactions with other immune cell populations in Mtb infection; we also discuss how host immunomodulation through DCs in response to Mtb and its products affects susceptibility and how innate and adaptive immunity is regulated by different types of DCs in Mtb infection.

## General roles of dendritic cells in Mtb infection

DCs bridge innate and acquired immunity. In a steady-state, DCs are derived from hematopoietic bone marrow progenitor cells and are present in an immature state in most tissues to detect and uptake foreign pathogens and their products. In an inflammatory environment, monocyte-derived DCs (moDCs) can differentiate *in situ* from monocytes (Hespel and Moser, 2012). As TB is a chronic disease in which the disease status alters the frequency of DCs. Patients with TB have fewer myeloid and plasmacytoid DCs (pDCs) in their peripheral blood than healthy controls (Uehira et al., 2002; Lichtner et al., 2006). After antibiotic treatment, the absolute number of pDCs was recovered; however, the number of myeloid DCs was not restored (Lichtner et al., 2006), indicating that DCs are involved in immunological changes in TB pathogenesis.

Once DCs detect and phagocytose pathogens or Ags, they undergo a maturation process, increasing the expression of MHC class I and II molecules, costimulatory molecules (CD80, CD86, and CD40), and chemokine receptor 7 (CCR7) to drive effective immunity (Mellman and Steinman, 2001). These phenotypic changes enable DCs to migrate toward the dLNs and effectively educate T cells. In an animal Mtb-challenge model, IL-12p40 deficient mice did not activate CD4^+^ T cells after Mtb infection and exhibited poor migration in response to the CCR7 ligands CCL19 and CCL21. However, IL-12p40 deficient DCs activated CD4^+^ T cells *in vitro*, where DCs do not need to migrate. The migration ability of DCs was recovered by additional IL-12p40 treatment (Khader et al., 2006). An analysis of 5530 patients with pulmonary TB and 5607 healthy controls showed that the DC migration regulator, ArfGAP with SH3 domain, ankyrin repeat, and PH domain 1 (ASAP1), was associated with susceptibility to TB (Curtis et al., 2015; Waltl, 2015). Once DCs arrive in local dLNs, they successfully present Ags to T lymphocytes with the molecules described above helping T cell activation, and induce effective cell-mediated immunity (Tascon et al., 2000; Marino et al., 2004). However, compared with other infectious diseases, the accumulation of Mtb-specific CD4^+^ T cell response in the lungs is delayed in Mtb-infected mouse models from two weeks post-infection (Reiley et al., 2008; Wolf et al., 2008), which is related to the obstruction of Ag presentation by Ag-presenting cells (APCs) such as DCs (Harding and Boom, 2010; Urdahl, 2014; Srivastava et al., 2016). Thus, the mechanical reasons for the delayed T cell response are dependent on how fast DCs interact with and recognize Mtb and its products. In addition, the combination of the Ag and DC subset that initially interact is important in inducing host-protective immunity.

Mtb infection induces a bacteria-favoring environment by regulating DC differentiation and function. In patients with TB, Mtb regulates the differentiation of DCs into the CD14^+^ moDC subset, which has a weak IL-12p70-producing capacity (Súndergaard et al., 2014). The Mtb-promoted CD14^+^ moDC subset induced a suboptimal T cell response, IL-17A-producing CD4^+^ T cells, rather than IFN-γ producing CD4^+^ T cell response (Súndergaard et al., 2014). Similarly, the generation of human monocyte-derived DCs with Mtb infection decreased CD80-expressing IL-12 and increased IL-10 secretion patterns, while CD1 expression that induces CD1-restricted T cell activation was inhibited (Gagliardi et al., 2007). In the same study, treatment with mycobacterial cell wall alpha-glucan elicited the Mtb-induced altered differentiation of DC (Gagliardi et al., 2007). Myeloid DCs and pDCs exhibited higher expression of B and T lymphocyte attenuator (BTLA), an immune inhibitory receptor, in patients with active TB than in healthy controls (Wang et al., 2017; Zhang et al., 2020). BTLA-positive myeloid DCs in patients with TB showed increased CCR7 expression, decreased IL-12 secretion, and decreased CD80 and CD83 expression. In addition, this DC subset showed a poor ability to uptake Ags and activate allogeneic T cell response but promoted Th2 and regulatory T cell response by secreting IL-4 and TGF-β (Zhang et al., 2020). Below, we review the major DC subsets involved in TB protection and pathogenesis.

## Dendritic cell classification and their interaction with Mtb

The classification of DC subset tends to be complex due to the lack of clear-cut differences in functionality, differences in markers depending hosts, and different properties depending on differentiation environment (*in vitro* or *in vivo*). Furthermore, classifications according to functionality such as inflammatory DCs and tolerogenic DC, make it more complex. DC subsets, previously classified by various nomenclatures, have been reclassified into a recently proposed simplified nomenclature based on ontogeny and function (Guilliams et al., 2014; Eisenbarth, 2019; Anderson et al., 2021). DCs can be divided into moDCs, conventional DCs (cDCs), and pDCs (Guilliams et al., 2014; Eisenbarth, 2019; Anderson et al., 2021). cDCs can be further divided based on surface molecules and transcription factors into type 1 cDCs (cDC1) and type 2 cDCs (cDC2). These DC subsets are differentiated into each subset through a slightly different process. In this model, multipotent progenitor differentiated from hematopoietic stem cells can be differentiated into macrophage DC progenitor (MDP). pDC is differentiated through pre-pDC populations derived from CDP or common lymphoid progenitor (Rodrigues et al., 2018; Dress et al., 2019). In addition, Feng et al. confirmed that pDC and cDC, especially cDC1, have a close relationship in development by using the FlipJump system (Feng et al., 2022). The differentiation of moDC occurs through common monocyte progenitor (cMop) differentiated from MDP rather than common DC progenitor (CDP). In tissues, Ly6C^+^ monocytes can be differentiated into cells functioning as macrophages or DCs (Mildner et al., 2013). The process through which monocytes differentiate into DCs is regulated by the concentration of PU.1, which suppresses the activity and expression of MafB, a macrophage transcription factor (Mildner et al., 2013; Menezes et al., 2016).

The cDC1 subset is IRF8- and BATF3-dependent and expresses the chemokine XC receptor 1 (XCR1) (Dorner et al., 2009; Bachem et al., 2010; Crozat et al., 2010) in humans and mice. In addition, cDC1 expresses different surface molecules such as CD8α, Dec-205, or CD103 in mice, and CD141 in humans (Edelson et al., 2010; Poulin et al., 2010; Satpathy et al., 2012; Williams et al., 2013) depending on tissues and organs. cDC2s are interferon regulatory factor 4 (IRF4)-dependent and can be identified by the surface marker CD11b (in humans and mice) along with different surface markers, such as DC immunoreceptor (DCIR) -2, CD301b, CD4, or signal regulatory protein-α (SIRPα) in mice, and CD1a in humans (Vremec et al., 2000; Suzuki et al., 2004; Satpathy et al., 2012; Schlitzer et al., 2013; Williams et al., 2013; Calabro et al., 2016). pDC is E2-2 dependent and expresses B220 and Siglec-H in mice and HLA-DR^+^CD123^+^CD303^+^ in humans (Cox et al., 1999; Onai et al., 2007; Poulin et al., 2010). moDC expresses CD11b and migrates to the inflammation site in a CCR2-dependent manner (Eisenbarth, 2019), and the differentiation of moDC is regulated by key transcription factors KLF4 and MAFB (Goudot et al., 2017; Jurkin et al., 2017). In particular, CD11b is expressed on both moDC and cDC2; therefore, distinguishing between the two subsets could be difficult in studies that have not used sufficient markers to discriminate the DC subsets.

It has been reported that DC subsets exhibit different properties and play various roles in Mtb infection. However, the functions of individual DC subsets remain controversial and require a detailed study. In Mtb-infected mice, both cDC1 and cDC2 are widely distributed and can be found in lymph nodes, blood and mucous membranes and migrate to the lung upon infection; however, after Mtb infection, CD103^+^ cDC1 is present in parenchyma and lung airways, with migratory ability to the dLNs (Geurtsvankessel et al., 2008; Geissmann et al., 2010; Guilliams et al., 2013; Leepiyasakulchai et al., 2013; Anderson et al., 2014). Similar to CD103^+^cDC1, CD8^+^cDC1 are primarily located in LNs. Koh et al. reported that CD103^+^ cDC1 has functions in constructing adaptive immunity, especially in the early stages of infection, by transporting bacteria to dLNs (Koh et al., 2017). However, CD11b^+^ Ly6C^low^ and CD11b^+^Ly6C^hi^ moDCs are primarily located in the lung parenchyma and LNs during Mtb infection (Mayer-Barber et al., 2011; Anderson et al., 2014; Norris and Ernst, 2018). In addition, CD11b^+^ DCs have been reported as the major subset harboring Mtb and migrating to the LNs of Mtb-infected mice (Wolf et al., 2007). Lai et al. reported that CD11b^+^ cDC2 is involved in protective immunity (Lai et al., 2018). CD103^+^ cDC1 inhibited CD11b^+^ DC-induced Th1 cell proliferation by secreting IL-10 (Lai et al., 2018). In contrast, other authors reported that CD103^+^ cDC1 is the main subset involved in the induction of protective response, secreting IL-12 (Leepiyasakulchai et al., 2013), inducing Th1 and Th17 responses (Sërgio et al., 2015), and restraining excessive inflammation through the recruitment of Tregs (Leepiyasakulchai et al., 2013). In addition, the adoptive transfer of CD103^+^DCs pulsed with Ag85B peptide significantly boosted *Mycobacterium bovis* bacillus Calmette-Guérin (BCG)-vaccinated mice with enhanced Th1 and Th17 responses (Griffiths et al., 2016). Meanwhile, failure to recruit CD103^+^ DCs diminished CD4^+^FoxP3^+^ regulatory T cells, resulting in increased Mtb susceptibility and excessive lung inflammation (Leepiyasakulchai et al., 2012). These studies showed that there is still controversy over the function of DC subsets and indicated that DC subsets could have different functions in Mtb infection, depending on the infection stage and environment.

Despite recent studies on the role of type I IFN in the TB pathogenesis (Moreira-Teixeira et al., 2018), studies on pDCs, one of the major sources of type I IFN, have not yet drawn much attention compared to other DC subsets. Studies on pDCs in TB have primarily focused on their frequency. Indirect evidence has been reported to suggest that pDC could be recruited to the infection site both in mice (Kim et al., 2015) and humans (Lu et al., 2017; Dirix et al., 2018). In addition, pDCs cooperate with Mtb-infected CD1c^+^ DCs, promoting the stimulation of CD4^+^ T cells in the LNs of TB patients (Lozza et al., 2014; Donovan et al., 2017). Despite the documented roles of DC subsets in Mtb infection across species, further studies are required to determine whether individual DC subsets with their unique features are drivers of host-protective or pathological immunity. In addition, these controversial outcomes should be considered along with the interactions of DCs with other cellular compartments in lung environments according to the Mtb infection stage.

## DC interaction with various cells

DCs play a sentinel role as APCs against pathogen invasion and induce an adaptive immune response *via* Ag presentation. This process is not unilateral but is regulated by bidirectional interactions with various cell populations. In this section, emerging evidence on the interaction and biological processes between DCs and innate and adaptive immune cells is reviewed with respect to the protective or pathological outcomes of TB.

### DC interactions with adaptive immune cells in Mtb infection

The IFN-γ produced by CD4^+^ T cells is considered a principal driver of host-protective immunity against TB (North, 1973; Shimokata et al., 1986; Orme et al., 1993). IFN-γ can activate macrophages promoting bactericidal ability The CD4^+^ T cell response could be primed with Ag presentation by DCs with MHC class II molecules (Chen and Kolls, 2013). In addition, IL-12p70 produced by Mtb-infected DCs is a key upstream cytokine that induces Th1 response (Cooper et al., 2007). Moreover, Mtb-infected DCs can promote the protective Th17 response against highly virulent Mtb infection by secreting IL-23, IL-6, and IL-1β (Gopal et al., 2014).

**Figure 1:**
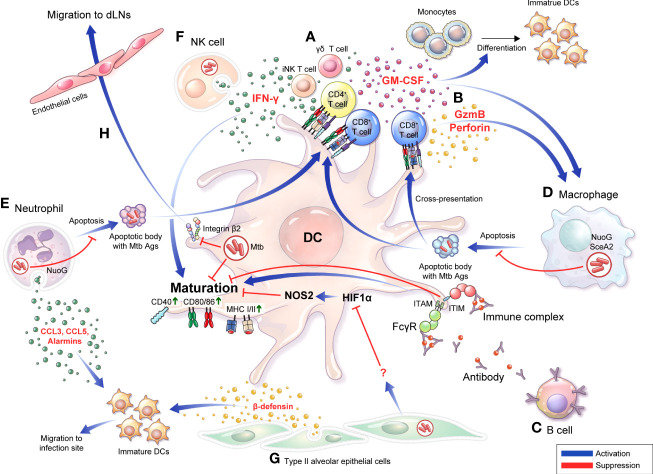
Bidirectional interactions between DCs and diverse cells are involved in the TB pathogenesis and protective response DCs do not function in the unilateral direction of pathogen uptake-migration-Ag presentation interaction with T cells. Bidirectional interactions between DCs and diverse cells are involved in the TB pathogenesis. **(A)** DCs secrete IL-12 that induce a Th1 immune response secreting IFN-γ or GM-CSF. Conversely, IFN-γ derived from activated T cells **(A)** and NK cells **(F)** can induce DC activation, and differentiation into mature DCs can be promoted. **(B)** CD8^+^ T cells are activated by DCs to secrete granzyme B or perforin, and CD8^+^ T cells simultaneously induce apoptosis of infected cells such as macrophages, thereby enabling effective Ags uptake by DCs. **(C)** IgG-produced B cells can bind to specific Ags, resulting in the formation of immune complexes. The function of DCs is affected by whether Abs or immune complexes bind to the inhibitory or activating Fcγ receptors with varying binding affinity depending on their isotype. **(D)** Apoptosis of macrophages is suppressed by NuoG or SecA in an Mtb-dependent manner, resulting in effective Ag presentation that could be suppressed, thereby suppressing T cell activation. **(E)** Mtb-infected neutrophils secrete alarmins, CCL3, and CCL5 through degranulation to promote migration of immature DCs to the infection site, DC migration to LNs, and induce maturation. In contrast, Mtb inhibits neutrophil apoptosis in a NuoG-dependent manner, thereby preventing this protective response. **(F)** In NK cells, DC maturation can be induced through IFN-γ secretion. **(G)** Alveolar epithelial cell type II secretes β-defensin to induce the migration of immature DCs to the infection site, and simultaneously regulates the DC Hif1α-NOS2 axis to induce DC maturation. **(H)** DCs expressing integrin β2 bind to endothelial cells and transmigrate to afferent lymphatic vessels. Mtb infection disturbs the expression of CD18 containing integrin β2, decreasing DC migration to local lymph nodes. GM-CSF, granulocyte-macrophage colony-stimulating factor; GzmB, granzyme B; FcγR, Fc Gamma Receptors; ITIM, immunoreceptor tyrosine-based inhibitory motif; ITAM, immunoreceptor tyrosine-based activation motif; dLN, draining lymph node.

Granulocyte-macrophage colony-stimulating factor (GM-CSF) is a major factor for the differentiation and homeostasis of DCs (Van De Laar et al., 2012). GM-CSF^−/−^ mice are highly susceptible to Mtb infection (Gonzalez-Juarrero et al., 2005; Szeliga et al., 2008). It has been recently reported that GM-CSF produced and secreted by T cells in Mtb-infected mice from 3 weeks after Mtb infection, and it can mediate protection *in vivo* (Rothchild et al., 2017). iNKT and γδT cells, unconventional T cells with innate responsiveness, in the early phase of Mtb infection, and CD4^+^ T cells after the third week of infection were the major sources of GM-CSF (Rothchild et al., 2017). In addition, the continuous production of GM-CSF by conventional T cells (Rothchild et al., 2017) may promote the differentiation of moDCs. Moreover, moDCs initiated acquired immune responses in the early stages of infection and took up Mtb-Ags, continuously moving in and out of granulomas to induce a protective T cell response (Harding et al., 2011; Schreiber et al., 2011a; Schreiber et al., 2011b). Therefore, the generation of GM-CSF by T cells in Mtb infection plays an important role in maintaining a continuous protective immune response through DC generation (**Figure 1A**).

CD8^+^ T cells are considered less critical for protection against Mtb infection than CD4^+^ T cells; however, accumulating emerging evidence has indicated the importance of CD8^+^ T cell response in protecting against TB (Chen et al., 2009; Nunes-Alves et al., 2014; Silva-Sanchez et al., 2015). Like CD4^+^ T cells, CD8^+^ T cells can produce IFN-γ, TNF-α, and IL-2, and exhibit an additional cytolytic function that kills Mtb-infected cells by producing perforin, granzyme, and granulysin (not in a mouse model), and it could induce apoptosis of Mtb-infected cells through Fas-Fas ligand interactions (Watson et al., 2000). The depletion of CD8^+^ T cells in Mtb-infected mice allows the uncontrolled growth of Mtb bacilli (Flynn et al., 1992; Mogues et al., 2001). In addition, increased CD8^+^ T cell depletion resulted in more detrimental outcomes in latent infections with antibiotic treatment than in acute infection mouse models (Van Pinxteren et al., 2000). In mice lacking perforin, one of the bactericidal apparatuses of CD8^+^ T cells, the cytolytic effect was decreased *in vivo*, and adoptive transfer of wild-type CD8^+^ T cells showed protective efficacy against Mtb infection (Woodworth et al., 2008). It is difficult to find human disease models lacking CD8^+^ T cells, but the depletion of CD8 cells in rhesus macaque (*Macaca mulatta*) displayed a significant decrease in protection against Mtb infection in BCG-vaccinated and reinfection models (Chen et al., 2009). CD8^+^ T cells can be primed by MHC class I molecules loaded with antigenic peptides, generally in the cytosol. This peptide-loading process could be due to the egression of Mtb or Mtb-Ags into the cytosol (Van Der Wel et al., 2007; Kaufmann, 2013) or phagocytosis of apoptotic vesicles of Mtb-infected DCs or macrophages by DCs (Schaible et al., 2003; Winau et al., 2006). In addition, after recognizing antigenic peptide displayed by DCs, CD4^+^ T cells release IFN-γ, whereas CD8^+^ T cells preferentially lyse APCs and recognize heavily infected DCs (Lewinsohn et al., 2003). In addition, the immunization of mice with DCs pulsed simultaneously with CD4, and CD8 peptides showed increased protection against Mtb infection but not immunization of DCs pulsed with CD4 peptide alone (Mcshane et al., 2002). These studies show that CD8^+^ T cells are important in inducing protective immune response through DCs against Mtb infection (**Figure 1B**). However, Mtb is also capable of interfering with CD8^+^ T cell priming by regulating the interaction of DCs through various mechanisms as will be discussed later.

Although T cell-mediated cellular immunity has been accepted as the major immune response to protect the host from Mtb (Nunes-Alves et al., 2014), evidence indicating the importance of B cell-mediated humoral immunity has also been accumulating. B cells are found in the lymphocytic cuff of human granulomas (Ulrichs et al., 2004; Tsai et al., 2006), and patients with TB show significant changes in B cell-associated genes after TB treatment (Cliff et al., 2013). In B cell-deficient mouse models, the administration of high-dose Mtb (Vordermeier et al., 1996; Maglione et al., 2007) induced higher bacterial loads compared to controls. In the same study, B cell-deficient mice showed neutrophilic inflammation and an upregulated Th17 response to Mtb infection (Maglione et al., 2007), suggesting that B cells could affect the disease outcome of Mtb infection by regulating inflammatory responses. Notably, B cells can modulate the inflammatory responses in DCs and can regulate the maturation, migration, and functional processes of DCs by producing cytokines (Skok et al., 1999; Kaser et al., 2000; Gonnella et al., 2001), chemokines (Xu et al., 1996; Lin et al., 1998; Crowley et al., 1999; Krzysiek et al., 1999), and antibodies that bind to the Fc receptor of APCs. DCs express Fc receptor (FcR) binding to the Fc region of antibodies, which is divided into stimulatory and inhibitory, depending on their intracellular, immunoreceptor tyrosine-based activation (ITAM), or immunoreceptor tyrosine-based inhibitory motifs (ITIMs) (Ravetch and Bolland, 2001; Nimmerjahn and Ravetch, 2006). FcR engagement can be strongly influenced by the antibody (Ab) isotype (Granstrøm et al., 1992). Therefore, the activation of DCs can be regulated through FcR by antibodies or immune complexes formed by antibodies, depending on the type of FcR engaged (Regnault et al., 1999; Geissmann et al., 2001; Schuurhuis et al., 2002; Bãnki et al., 2003; Dhodapkar et al., 2005). In an Mtb-challenged mouse model, an inhibitory FcR, Fc gamma receptor IIB (FcγRIIB) deficiency reduced Mtb growth and immunopathology compared with WT mice. In the same study, Fcγ RIIB-deficient mice showed increased IFN-γ-producing CD4^+^ T cells and elevated MHC class II expression, costimulatory CD80, and CD86 in the lungs (Maglione et al., 2008). These studies show potential for the interaction between DCs and B cells *via* Abs (**Figure 1C**). Thus, understanding how DCs modulate host immune responses by interacting with T and B cells is critical for developing anti-TB vaccines.

### Interactions between DCs and innate immune cell compartments in Mtb infection

In addition to interacting with T cells, DCs, directly and indirectly, interact with various types of immune cells, thereby affecting the outcomes of Mtb infection. Macrophages are major host cells involved in the invasion, growth, and restriction of Mtb in infected hosts. Mtb-infected macrophages undergo apoptosis, and efferocytosis of apoptotic macrophages by DCs promotes acquired immunity. After phagocytosis of apoptotic macrophages, DCs can present Mtb-Ags through the cross-presentation pathway, leading to activating CD8^+^ T cells *in vitro* (Schaible et al., 2003; Weiss and Schaible, 2015; Sia et al., 2017). Macrophages infected with *secA2*-deleted Mtb promoted macrophage apoptosis by decreasing mycobacterial superoxide dismutase (Hinchey et al., 2007). Increased apoptosis by deleting Mtb *secA2* induces significant priming of CD8^+^ T cells in *in vivo* mouse models (Hinchey et al., 2007).

Similarly, Velmurugan et al. reported that *nuoG* of Mtb, which encodes a subunit of the type I nicotinamide adenine dinucleotide (NADH) dehydrogenase complex, inhibits Mtb-infected macrophage apoptosis, and the infection of macrophages with the *nuoG-*deleted Mtb mutant induced increased macrophage apoptosis *in vitro* and increased macrophage survival with controlled bacterial burden and lung pathology in *in vivo* mouse studies (Velmurugan et al., 2007) (**Figure 1D**). Recently, it was confirmed that a single-nucleotide polymorphism of Siglec-1 (CD169), a cell adhesion and endocytic receptor that recognizes sialylated glycans, was correlated with extrapulmonary dissemination of Mtb (Benet et al., 2021), susceptibility to Mtb infection, and activation of pulmonary TB in human cohort studies (Souza De Lima et al., 2017). Mtb infection induced the local spread of bacteria within the lung of CD169 deficient mice, resulting in more extensive pathogenic lesions than wild-type mice. In the same study, human DCs activated T cells by the uptake of extracellular vesicles purified from Mtb-infected THP-1-derived macrophages in a CD169 dependent manner (Benet et al., 2021). It has been recently reported that CD169 on macrophages preferentially binds to and interacts with CD8α^+^ cDCs for CD8^+^ T cell cross-priming (Van Dinther et al., 2018), suggesting the importance of the interaction between DCs and macrophages in CD8^+^ T cell immunity formation in TB pathogenesis.

The role of neutrophils in TB pathogenesis remains controversial (Lowe et al., 2012; Kroon et al., 2018), and studies on the role of neutrophils and their relationship with other immune cells are continuing for more accurate identification. Abundant neutrophils are observed in the bronchoalveolar lavage fluid of patients with pulmonary TB (Law et al., 1996) and are the most commonly infected phagocytes in patients with TB (Eum et al., 2010). At the site of inflammation, neutrophils secrete chemokine profiles, such as the induction of CCL3 and CCL5 (Scapini et al., 2000) and alarmins during degranulation (Yang et al., 2000), which contributes to the chemoattraction of immature DCs. In addition, DCs directly infected by Mtb showed a poor response to CCL19 in migration experiments, whereas DCs that had acquired Mtb through uptake of infected neutrophils showed unimpaired migration capacity that facilitates the initiation of CD4^+^ T cell response (Blomgran and Ernst, 2011). In this line, neutrophil-depleted mice with Mtb infection showed decreased DC trafficking in the mediastinal LNs (mLNs), resulting in delayed activation and proliferation of Ag85B-specific CD4^+^ T cells in mLNs (Blomgran and Ernst, 2011). Neutrophils also interact with DCs during BCG infection to induce protective immunity. Morel et al. reported that non–apoptotic BCG-infected neutrophils clustered with immature DCs, establishing intimate contact with DC dendrites *in vitro*; this physical interaction induced DC activation in humans and mice (Morel et al., 2008). In addition, BCG-infected neutrophils decreased IL-10 secretion in human DCs, sustained secretion of IL-2 in mouse DCs, and promoted CD4^+^ T and CD8^+^ T cell proliferation by promoting Ag presentation of DCs, suggesting that neutrophils promote Ag-cross-presentation of DCs (Alemãn et al., 2007; Morel et al., 2008). Mtb-induced neutrophil apoptosis induced the functional maturation of DCs (Hedlund et al., 2010). However, Blomgran et al. reported that Mtb delayed T cell response by inhibiting the ability of DCs to act as APCs *via* Mtb-infected neutrophil-apoptosis inhibition (Blomgran et al., 2012). However, similar to macrophages (Velmurugan et al., 2007), infection with Mtb mutants lacking *nuoG* reduced neutrophil life span with the acquisition of fewer Mtb per neutrophil, induced earlier Mtb infected-DC migration to LNs, resulting in the acceleration of CD4^+^ T cell priming. However, neutrophil depletion in mice infected with Mtb mutants lacking *nuoG* reduced priming of CD4^+^ T cell, suggesting that the inhibited apoptosis of neutrophil delayed adaptive immunity in TB (Velmurugan et al., 2007) (**Figure 1E**). Collectively, DC-neutrophil interaction has a role in protective immunity in TB.

Natural killer (NK) cells are observed at the site of infection immediately after Mtb infection (Junqueira-Kipnis et al., 2003), and are increasingly recognized as a key component of the innate immune response linking innate and adaptive immunity (Gabrielli et al., 2016; Choreño Parra et al., 2017). NK cells are potent producers of IFN-γ that promote DC maturation and stimulate naïve T cell differentiation into Th1 cells (Pan et al., 2004; Frasca et al., 2008; Ferlazzo and Morandi, 2014). In Mtb infection, the cytolytic activity of NK cells freshly isolated from human PBMCs was strongly augmented by co-culture with Mtb-infected DCs, and NK cells reciprocally enhanced DC maturation and IL-12 production (Gerosa et al., 2002). Moreover, BCG-vaccinated mice with NK cell depletion showed fewer activated DCs which in turn reduced the frequency of IFN-γ producing CD4^+^ T cells in the lungs and spleen (Junqueira-Kipnis et al., 2020). These results indicated that NK cells influence adaptive immune responses through interactions with DCs (**Figure 1F**).

### Interactions between DCs and non-immune cell populations in Mtb infection

Type II alveolar epithelial cells (AEC-II) can be infected by Mtb and provided as a niche (Ryndak et al., 2015). However, it has been recently reported that Mtb-infected AEC-II interacts with and modulates DC function. Mtb-infected AEC-II indirectly induced DC maturation by negatively regulating HIF-1α induced NOS2 and switching DC metabolism (Rodrigues et al., 2020). In addition, beta defensin-2 can be produced by Mtb-infected human AEC-II (Rivas-Santiago et al., 2005) and recruits immature DCs to the infection site by binding to the DC chemokine receptor CCR6 (Yang et al., 1999; Biragyn et al., 2002). These results suggest that AEC-II affects the recruitment of DCs to the infection site at the beginning of Mtb infection (**Figure 1G**).

After Mtb phagocytosis, DCs migrate from the lung to local LNs through lung endothelial cells, and Mtb-infected human DCs show reduced expression of CD18-containing cell surface integrins (Roberts and Robinson, 2014). These molecules regulated the adhesion and transmigration of DCs through endothelial cells (D’amico et al., 1998; De La Rosa et al., 2003). Consistent with reduced integrin surface expression, Mtb-infected DCs displayed a significant reduction in adherence to lung endothelial cells and migration toward lymphatic chemokines (Roberts and Robinson, 2014) (**Figure 1H**).

There are remain many gaps in our understanding of the interaction between DCs and other cells. The fact that DCs interact with various cells means that the immune evasion mechanism of Mtb for a specific cell can also affect the interaction of the cell with DCs. However, existing studies are the results of observations in a specific experimental situation or specific disease state. Since the interaction between DC and other cells may exhibit different aspects depending on conditions such as location or disease state, therefore an integrated study is required. Understanding the crosstalk between cells could provide a new perspective on TB control. Furthermore, considering cell-cell interactions, vaccine development and HDTs could improve TB control.

## New perspective on the role of DCs in inducible bronchus-associated lymphoid tissue and germinal center formation in Mtb infection

While secondary lymphoid organs have specific locations for the immune response, a chronic immune response could induce organized accumulations of lymphoid cells similar to those of secondary lymphoid organs in non-lymphoid tissue. These tertiary lymphoid organs have structures similar to secondary lymphoid organs, especially LNs, and are identified in chronic inflammatory processes, such as cancer (Bergomas et al., 2011; Martinet et al., 2011), chronic infection (Drayton et al., 2006), and atherosclerosis (Græbner et al., 2009). Inducible bronchus-associated lymphoid tissue (iBALT) is a tertiary lymphoid structure that resembles secondary lymphoid structure that is found in various pulmonary infectious diseases, including TB, and has been suggested to play a role in protection (Silva-Sanchez and Randall, 2020). The iBALT structure can maintain a locally activated Ag-specific lymphocyte pool that elicits a rapid and effective immune response (Moyron-Quiroz et al., 2004; Jones and Jones, 2016).

iBALT formation in Mtb infection correlates with protection. iBALT induction is mediated by CXCL13 (Khader et al., 2009; Rangel-Moreno et al., 2007; Rangel-Moreno et al., 2011; Khader et al., 2011) that controls the formation of B cell follicles, T cell placement, and optimal macrophage activation for Mtb control (Khader et al., 2009; Khader et al., 2011). Mtb infection induces the formation of granulomas characterized by a central core of infected macrophages surrounded by lymphocytes. Granuloma can isolate Mtb to inhibit growth, and at the same time can act as a shelter for Mtb against host immunity (Cadena et al., 2017). iBALT is found in the perivascular space along the airways of the lung, enabling rapid protective immune response (Hwang et al., 2016; Fleige and Førster, 2017). In a nonhuman primate model (Kaushal et al., 2015) and patients with TB (Ulrichs et al., 2004; Ulrichs et al., 2005), the presence of B cell follicles correlates with protection in TB (Kaushal et al., 2015). In addition, the presence or absence of iBALT was associated with the maintenance of latent infection or the development of active disease (Ulrichs et al., 2004; Slight et al., 2013). The use of alternative vaccination routes, such as mucosal or intravenous, leads to the generation of iBALT, which has been associated with reduced bacterial burden (Perdomo et al., 2016; Christensen et al., 2017; Counoupas et al., 2020; Darrah et al., 2020). The iBALT structure is maintained for a specific time even after the inflammatory response is over (Rangel-Moreno et al., 2011; Morissette et al., 2014), and considering the characteristics of iBALT, it may provide a site for the localization of protective lymphocytes such as CXCR5^+^ CD4^+^ T cells, which correlate with a better prognosis for patients with TB (Khader et al., 2011; Slight et al., 2013). Although the exact mechanism by which the TB vaccine-derived IBALT induces a protective effect has not yet been precisely elucidated, it would be a reasonable goal to develop a vaccine that induces iBALT, considering its protective effect in TB.

Follicular dendritic cells (FDCs) are non-hematopoietic cells of stromal origin (Krautler et al., 2012) that play an important role in B cell activation and bind and retain Ags in B cell hair follicles for long periods (Kranich and Krautler, 2016; Melzi et al., 2018). CCL19 and CCL21 are expressed by FDCs and recruit naïve, Ag-specific memory T cells and cDCs in the early T cell regions of iBALT (Moyron-Quiroz et al., 2004; Fleige et al., 2018). In addition, FDC-derived CXCL13 can induce the homing of CXCR5-expressing B and T cells to iBALT (Moyron-Quiroz et al., 2004). Previous iBALT studies have mainly focused on the functions of FDCs that induce germinal center formation in secondary lymphoid organs; however, several studies have reported that traditional DCs affect the formation and maintenance of iBALT in inflammation. It has been reported that *Bartonella henselae*-infected mouse bone marrow-derived dendritic cells and lung DCs produce CXCL13, which is essential for iBALT formation (Vermi et al., 2006). In addition, DCs are necessary to maintain iBALT in response to viral infection in mouse models (Geurtsvankessel et al., 2009; Halle et al., 2009). Pulmonary delivery of Mtb Ag-primed DCs rapidly increases iBALT formation and near-bactericidal immunity and improves the disease outcome (Griffiths et al., 2016). These studies indicate that the relationship between iBALT and DCs in TB control is important and requires further research. Furthermore, it is possible to induce and maintain protective immunization with vaccines by understanding and controlling the iBALT formation and maintenance mechanisms through DCs.

## Interaction between Mtb and its components with DCs

**Figure 2:**
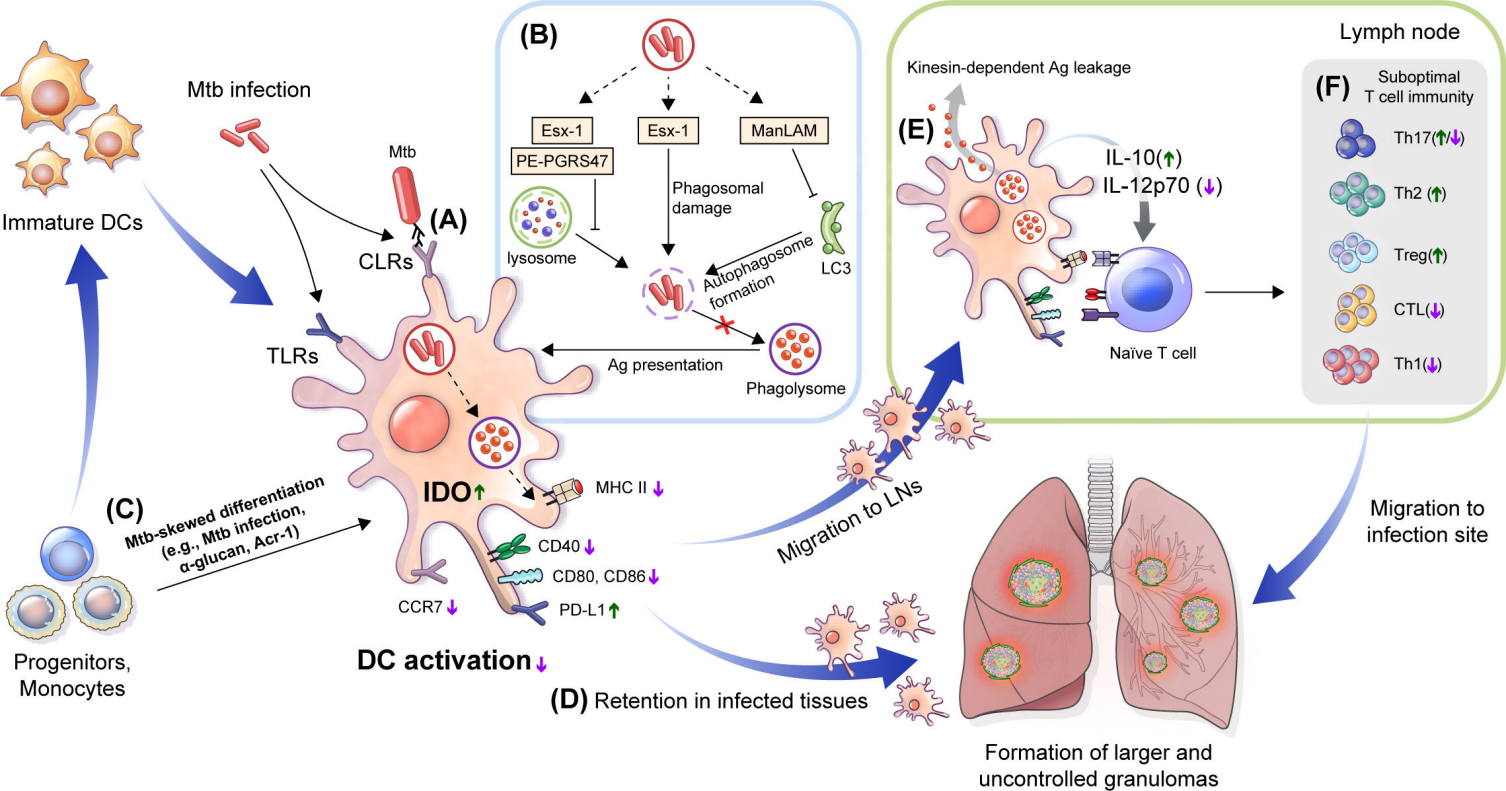
Immune alteration mechanisms of Mtb targeting DCs. **(A)** DC differentiation is affected by Mtb. Mtb-Ags such as Acr-1 or α-glucan, a cell wall component of Mtb, induce altered differentiation of DCs with reduced function. **(B)** Mtb, its cell walls components and Mtb-Ags are recognized by DCs *via* TLRs and CLRs, which could induce alteration of DC function by down regulating the expression of costimulatory molecules (CD80, CD83, and CD86) and MHC class II to suppress maturation, and increase the expression of inhibitory molecules such as PD-L1 and IDO. **(C)** Mtb inhibits Ag presentation. Esx-1 induces phagosomal damage and together with PE-PGRS47, inhibits phagosome-lysosme fusion. Meanwhile, ManLAM suppresses autophagosome formation by inhibiting expression of microtubule-associated light chain 3 (LC3) protein. **(D)** Reduced expression of CCR7 by Mtb infection affect DC migration to the LNs by lowering response to CCL19 and CCL21. DCs captured in lung tissue promote the formation of larger or multifocal granulomas. **(E)** DC migration to lymph nodes causes leakage of Mtb-Ags in a kinesin-2 dependent manner, and induces suboptimal T cell proliferation by the inefficient by Mtb-induced maturation. Cytokine profiles such as increased IL-10 and decreased IL-12p70 interfere with protective Th1 type polarization. **(F)** These processes induce a delayed T cell response to lung tissue infection sites, and suppress TB disease control by forming suboptimal T cell immunity. TLRs, Toll-like receptors; CLRs, C-type lectin receptors; PD-L1, programmed death-ligand 1; IDO, indoleamine 2,3-dioxygenase; LNs, lymph nodes; CCR, chemokine receptor; CCL, chemokine ligand.

As described above, DCs play diverse roles in controlling Mtb infection by interacting with other cellular compartments in Mtb infection. However, DCs may also be a simultaneous target of the Mtb host immune-evasion mechanism to generate a pathogen-favoring environment (**Figure 2**, **Table 1**). This section discusses the defined Mtb molecules and relevant pathways facilitating pathogenesis.

**Table 1:** Mtb and its components that inhibit DC function.

Factor	Mechanism	Consequence	Category	Ref.
Mtb infection	Decrease in expression of CCR7	- Promote lung granuloma dissemination - Reducing Ag availability	Migration	(Harding et al., 2015)
Mtb infection	Decrease in expression of CD18	- Limited Ag presentation to T cells in LNs	Migration	(Bhatt et al., 2004)
Mtb infection	Leakage of Ags in Mtb-infected DCs *via* kinesin 2-dependent vesicular transport	- Limit Ag presentation to T cells in LNs	Ag presentation	(Srivastava et al., 2016)
Zmp1	Arrest of phagosome maturation of DCs	- Increased Ag85A presentation by DCs infected with BCG *Zmp1*mutant compared to DCs infected with wild type BCG - Increased IFN-γ producing CD4^+^-/CD8^+^-T cells in BCG *Zmp1*mutant immunized mice.	Ag presentation	(Master et al., 2008; Johansen et al., 2011)
PE-PGRS47	Inhibition of autophagosome-lysosome fusion	- Enhanced MHC class II-restricted Ag presentation in mice infected with *PE-PGRS47* deficient Mtb	Ag presentation	(Saini et al., 2016)
ESX-1	Impairment of autophagosome-lysosome fusion	- Decreased IL-12 expression in DCs and impairment of Th1 response	Ag presentation	(Romagnoli et al., 2012)
EsxH	Inhibition of the endosomal sorting complex required for transport (ESCRT) machinery	- esxH-deficient Mtb induced more Mtb Ag-specific CD4^+^ T cell proliferation than wild type Mtb	Ag presentation	(Portal-Celhay et al., 2016)
Heat-killed Mtb prime boost vaccination	Induction of myeloid-derived suppressor cells (MDSCs)	- MDSCs produced NO, which killed DCs in spleen	Differentiation	(Ahn et al., 2012)
Acr-1	Impairment of DCs maturation	- Decreased induction of IFN-γ producing CD4^+^ T cells	Differentiation	(Amir et al., 2017)
ManLAMs	Promotion of IL-10 secretion, reducing IL-12 by binding to DC-SIGN on DCs	- Decrease in IFN-γ from T cell co-cultured with ManLAM stimulated DCs	Maturation and cytokines	(Geijtenbeek et al., 2003; Chieppa et al., 2003; Andersen and Doherty, 2005; Wu et al., 2011; Orme, 2013; Balboa et al., 2016; Andersen and Scriba, 2019)
Glycolipid Di-O-acyl trehalose	Decrease in IL-12 and increase in IL-10 and IDO	- Promoted expansion of FoxP3^+^ regulatory T cell	Maturation and cytokines	(Magallanes-Puebla et al., 2018a)
Rv1016c-overexpressing BCG (rBCG-Rv1016c)	Decreased the production of cytokines (IL-2, IL-12p70, TGF-β, IL-6) and co-stimulatory molecules (CD80, CD86, MHC class I, MHC class II)	- Impaired Th1 and Th17 responses	Maturation and cytokines	(Su et al., 2019)
Hip1	Decrease in IL-12, CD40, CD86, MHC class II molecules *via* MyD88- and TLR2/9-dependent pathways	- Impaired Th1 and Th17 responses	Maturation and cytokines	(Su et al., 2019)
GroEL2	Cleavage to monomer by Hip1 to inhibit DCs maturation (reduced CD40, CD86, IL-6, IL-12p40)	- Impaired Th1 and Th17 responses	Maturation and cytokines	(Georgieva et al., 2018)

CCR, chemokine receptor; LNs, lymph nodes; NO, nitric oxide; MDSCs, myeloid-derived suppressor cells; IDO, indoleamine 2,3-dioxygenase.

### Interaction of DCs with Mtb cell wall components

Various cell wall components of Mtb strains can modulate DC immune response. The cell wall of mycobacteria contains various glucoconjugates such as peptidoglycan, arabinogalactan, and glycolipids. Some of these glucoconjugates regulate host immune responses mainly by binding to C-type lectin receptors (CLRs), a family of pattern recognition receptors that include the mannose receptor (MR), DC-SIGN, Dectin-2, DCIR, and Mincle (**Figure 2A**). For example, mycobacterial glycolipid Di-O-acyl trehalose promoted IL-10 secretion and indoleamine 2, 3-dioxygenase (IDO) and downregulated IL-12 secretion and costimulatory molecule expression in DCs, promoting FoxP3^+^ regulatory T cell expansion (Magallanes-Puebla et al., 2018b). DCIR-deficient control mice exhibited better control of Mtb infection with increased TNF-α production and inducible NOS in its lungs compared to wild-type controls, supporting the immune regulatory mechanism of Mtb cell wall components *via* CLRs (Troegeler et al., 2017).

Another Mtb cell wall component, mannose-capped lipoarabinomannan (ManLAM), regulates DC activation, but the results are contradictory. Because of its structural complexity, ManLAM can be recognized by several receptors, such as MR, DC-SIGN (Pan et al., 2014), Toll-like receptor (TLR) 2 (Gilleron et al., 2006), DC immunoactivating receptor (DCAR) (Toyonaga et al., 2016), and mannose-binding protein (MBP) (Hoppe et al., 1997). Complement receptor (CR)3 and MR are major binding receptors of macrophages for Mtb (Schorey et al., 1997; Ernst, 1998), but they played a minor role in Mtb infection in moDC, rather DC-SIGN is a major binding receptor for Mtb infection (Tailleux et al., 2003). In the same study, Mtb binding through DC-SIGN in DCs was confirmed to occur in a lipoarabinomannan (LAM)-dependent manner, and it was confirmed by observing the binding of DC-SIGN^+^ lung DCs with Mtb in the LNs of patients with TB. ManLAM from virulent Mtb H37Rv induced the maturation of DCs and secretion of IL-6, IL-12 and TNF-α in human DCs (Mazurek et al., 2012), and promotes increased Ag presentation (Yonekawa et al., 2014). Subsequently, it has been reported that ManLAM-induced DC activation occurs via Dectin-2 (Yonekawa et al., 2014). Dulphy et al. reported that ManLAM induces intermediate human DC maturation (Dulphy et al., 2007). In contrast, ManLAM treatment of human moDCs inhibited lipopolysaccharide (LPS) mycobacteria-induced DC maturation, and DC maturation was restored when DC-SIGN was blocked (Geijtenbeek et al., 2003). ManLAM treatment also induces human DCs to increase IL-10 secretion, resulting in reduced IFN-γ secreting T cell response (Wu et al., 2011); furthermore, it inhibits IL-12 and promotes IL-10, IL-1R antagonist and IL-1R type II secretion and a similar DC maturation profile was observed in MR-specific Ab-treated DCs (Chieppa et al., 2003). ManLAM-induced DC maturation inhibition was reversed by blocking its interaction with the MR with the ssDNA aptamer ZXL1, resulting in increased T cell activation (Pan et al., 2014). These discrepancies in the effects of ManLAM on DCs may be due to the structural differences in ManLAM from various bacterial strains (Kællenius et al., 2016) or the complexity of the receptor recognized by ManLAM, indicating that further studies are required.

Mycobacterial lipid Ags are derived from the cell wall. CD1 molecules have a unique ability to present lipid Ags. The CD1 family can be classified by its recognition of Ags presented by group 1 CD1 molecules (CD1a, CD1b, and CD1c) or by CD1d. DCs express CD1 and can interact with CD1-restricted CD8 T cells, αβ T cells, γδ T cells, and NK cells (Rosat et al., 1999; Schaible et al., 2000; Fischer et al., 2004). These lipid Ags include mycolic acid (Beckman et al., 1994), glucose monomycolate (Moody et al., 1997), lipoarabinomannans (Sieling et al., 1995; Torrelles et al., 2012), phosphatidylinositol mannosides (Ernst et al., 1998; Cala-De Paepe et al., 2012), glycerol monomycolate (Layre et al., 2009), and sulfoglycolipids (Gilleron et al., 2004). Lipid Ag-specific T cells primed through this unconventional Ag presentation could perform protective functions during Mtb infection (De Libero and Mori, 2014). Mtb inhibits the formation of a complex of MHC class II molecules and peptides in DCs during infection, but unconventional Ag presentation through CD1 can induce rapid Ag presentation and thus a CD1-restricted T cell response (Hava et al., 2008). However, Mtb can also induce immune evasion by inhibiting CD1 expression on DCs (Stenger et al., 1998). Recently, mycolic acid induced both humoral and cellular immunity in tumor vaccine model, and it could induced anti-tumor immune responses in tumor vaccination models as well as in therapeutic models by enhancing Ag-specific cytotoxic T cell activity, indicating a potential for lipid Ag as an adjuvant (Kubota et al., 2020).

### Interaction of DCs with Mtb protein Ags

Various Mtb-Ags have been explored as TB subunit vaccine targets, some of which can induce a protective immune response through DCs (**Table 2**). However, some Mtb-Ags modulate the DC immune response. For example, the Rv1016c protein, a virulence factor required for prolonged survival in macrophages (Gonzãlez-Zamorano et al., 2009), enhanced BCG virulence when overexpressed, impairing DC activation which in turn inhibited Th1 and Th17 differentiation (Su et al., 2019). In the same line, the serine hydrolase Hip1 (Madan-Lala et al., 2014) and monomeric GroEL2 cleaved by Mtb Hip1 (Georgieva et al., 2018) suppressed Th1 and Th17 T cell polarization by inhibiting DC maturation

**Table 2:** Ags of Mtb that induce DC activation.

Factor	Mechanism	Consequence	Category	Ref.
HSP70	Functioning as alternative CD40L, bind to CD40	- Increased IL-12, TNF-α, and NO expression - Induced DCs maturation	Maturation and cytokines	(Lehner et al., 2004)
PE_PGRS11 PE_PGRS17	TLR-2-mediated maturation and activation of human DCs	- Induced strong CD4^+^ T cell response and proliferation	Maturation and cytokines	(Bansal et al., 2010)
Rv0315	Increase DCs maturation (increased expression of CD80, CD86, MHC class I/II and secretion of IL-6, IL-1β, TNF-α)	- Induced Th1 polarization - Increased secretion of IFN-γ from splenic CD4^+^ T cell and CD8^+^ T cell	Maturation and cytokines	(Heo et al., 2011)
PstS1	Promotes DCs phenotypic activation and IL-6, IL-1β and IL-23 secretion in DCs	- Induction of IFN-γ and IL-17/IL-22 response of T cell	Maturation and cytokines	(Palma et al., 2013)
Rv3812	Increase DCs maturation (increased expression of CD80, CD86, MHC class II and secretion of IL-6, IL-1β, TNF-α)	- Increased IL-2 and IFN-γ of CD4^+^ T cell	Maturation and cytokines	(Vani et al., 2013)
RpfB	TLR-4 mediated maturation of DCs	- Polarized naïve CD4^+^ and CD8^+^ T cells to secrete IFN-γ and IL-2. - Induced the expansion of memory T cells in the spleen of Mtb-infected mice	Maturation and cytokines	(Kim et al., 2013)
Rv0577	TLR-2 mediated BMDCs maturation (increased expression of CD80, CD86, MHC class I/II and secretion of TNF-α, IL-1β, IL-6, and IL-12p70)	- Induced Th1 polarization - Increased secretion of IFN-γ from splenic CD4^+^ T cell and CD8^+^ T cell	Maturation and cytokines	(Byun et al., 2012)
Rv2220	Induced maturation of DCs mediated by MAPK and NF-κB signaling pathway	- Increased the expansion of CD62L^lo^ CD44^hi^ CD4 memory T cells in spleen of Mtb infected mice	Maturation and cytokines	(Choi et al., 2018a)
GrpE	Induced TLR-4 mediated maturation of DCs	- Induced the proliferation of GrpE-specific Th1-type effector memory T cells from the spleen of Mtb infected mice	Maturation and cytokines	(Kim et al., 2018)
PPE60	Induced TLR-2 mediated DCs maturation (increased expression of CD80, CD86, MHC class I/II and secretion of TNF-α, IL-1β, IL-6, IL-12p70, and IL-23p19)	- Increased secretion of IFN-γ and IL-17 from CD4^+^ T cell	Maturation and cytokines	(Su et al., 2018)
Rv3841	Induced TLR-4 mediated maturation of DCs (increased expression of CD40, CD80, CD86, MHC class II and secretion of TNF-α, IL-12p70)	- Induced the proliferation of Th1 cell - Increased the expansion of CD62L^lo^CD44^hi^CD4^+^ memory T cells in spleen of Mtb infected mice	Maturation and cytokines	(Choi et al., 2018b)

NO, nitric oxide; BMDCs, bone marrow-derived dendritic cells; MAPK, mitogen-activated protein kinase.

Some Mtb-Ags evade the host immune system by inhibiting the processing and presentation of Mtb-Ags interaction with T cells (**Figure 2B**). The 6 kDa early secretory antigenic target (ESAT-6), one of the major Mtb-Ags, inhibits human DC maturation and IL-12 production but promotes IL-23 and IL-1b secretion, which in turn promotes a Th17 rather than a Th1 response (Wang et al., 2012). Autophagy is a homeostatic mechanism that can participate in host defense as a multistep process involving the enclosing and lysing of intracytoplasmic cargo, such as Mtb, by merging with lysosomes and favoring antigen presentation. Autophagosome-lysosome fusion in human DCs was inhibited by infection with the virulent H37Rv strain through ESAT-6 secretion system-1 (ESX-1) activity (Romagnoli et al., 2012). This study suggests that Mtb suppressed autophagy, which is required for an efficient Ag presentation and subsequent T cell activation (Jagannath et al., 2009; Münz, 2009). The zinc metalloprotease 1 (Zmp1) of Mtb arrests DC phagosome maturation (Master et al., 2008), which limits the presentation of MHC class II-restricted Ags (Johansen et al., 2011). Ag presentation could be affected by Mtb protein PE-PGRS47, inhibiting the effective autophagosome-lysosome fusion by suppressing the autophagy pathway (Saini et al., 2016), and Mtb EsxH, inhibiting the endosomal sorting complex required for the transport (ESCRT) machinery required for Ag processing (Mehra et al., 2013; Portal-Celhay et al., 2016). These studies show that Mtb-Ags can evade the host immune system by inhibiting the Ag presentation of DCs. The latency-associated protein alpha-crystallin protein (Acr-1) of Mtb regulates DC function by regulating its differentiation stages (Siddiqui et al., 2014) (**Figure 2C**). Mouse DCs generated in the presence of Acr-1 displayed decreased expression of CD80, CD86, and MHC class II and increased expression of PD-L1, Tim-3, IDO, and IL-10, which promote regulatory T cell generation; however, DC generation with CFP-10 or ESAT-6 did not affect DC function and phenotype. These reports show that Mtb induces host immune evasion by impairing the function of DC, such as inhibition of the Ag presentation and alteration of differentiation.

Moreover, Mtb-infected DCs exhibit a low CCR7 expression level and migrate less efficiently than non-infected DCs (**Figure 2D**). Mtb-specific T cells may capture infected DCs in granulomatous tissue, which reduces Ag availability in dLNs and induces the retention of DCs in infected tissue (Harding et al., 2015), promoting the dissemination and formation of new or larger multifocal lesions (Harding et al., 2015). Although Mtb-infected DCs migrate to LNs, they have a poor ability to activate CD4^+^ T cells directly. In addition, Mtb-infected DCs export Mtb-Ags to bystander resident DCs in a kinesin-2 dependent manner, this is insufficient to compensate for the reduced Ag presentation by infected DCs (Srivastava et al., 2016) (**Figure 2E**). Mtb infection modulates overall DC function such as maturation, migration and Ag presentation to construct protective T cell immunity (**Figure 2F**). Mtb-Ags allow Mtb to evade host immunity by regulating the various functions of DC, suggesting that there is a possibility of additional mechanisms by unknown Ag, while suggesting that these Ags may be potential targets for TB control. Therefore, it is important to discover and identify Mtb-Ags that modulate DC function.

## The role of DC metabolism in TB

Metabolites are sensitively regulated by the immune response, and metabolic profiles have been applied as a biomarker in various diseases, such as sepsis, leprosy, and diabetes (Amaral et al., 2013; Langley et al., 2013; Langley et al., 2014; Tam et al., 2017). In the case of TB, differences in the metabolic profile of plasma or serum between TB patients and healthy controls have been reported using liquid chromatography high-resolution mass spectrometry (LC-MS) (Frediani et al., 2014; Feng et al., 2015). Recently, metabolic changes in blood have been reported as an index predicting the onset of TB disease in Sub-Saharan Africa (Weiner et al., 2018). These reports suggest a strong association between metabolism and the disease state of TB.

Immune response to disease state affects metabolic changes in various cells. In an early study, it was shown that changes in macrophage metabolism reflect macrophage activation (Hard, 1970), and since then, there have been reports that various immune responses affect metabolism in specific cells, such as DCs (Kominsky et al., 2010; Kelly and O’neill, 2015). To generate ATP under normoxic conditions, DCs can use oxidative phosphorylation (OXPHOS), but under hypoxia glycolytic metabolism is induced to generate ATP independently of OXPHOS. In addition to hypoxic conditions, stimulation of TLRs, such as LPS, simultaneously induces DC activation and aerobic glycolysis through metabolic reprogramming, which plays an important role in DC activation (Krawczyk et al., 2010; Everts et al., 2014). In addition, DC metabolism could be regulated by inflammatory conditions, such as TLR stimulation, as well as by the metabolic environment. Lawless et al. reported that, while human moDCs induced maturation *via* glycolysis in a restricted glucose environment, DC maturation was rather decreased in a high-glucose environment and decreased immunogenicity for T cell activation (Lawless et al., 2017). cDCs cannot produce nitric oxide (NO), whereas moDCs differentiated with GM-CSF can produce NO. Since NO is a strong inhibitor of the electron transport system which is critical for OXPHOS (Bailey et al., 2019), metabolic reprogramming of cDCs can be induced by NO from moDCs or macrophages (Everts et al., 2012). These reports suggest that the different functions of DCs depending on tissue localization during TB could be affected by the metabolic environments. For example, Mtb-infected DCs migrate to LNs, but direct interaction with T cells occurs by LN resident cDCs with Ags transferred from Mtb-infected DCs (Srivastava and Ernst, 2014). Since a significant proportion of DCs migrating to LNs are moDCs capable of producing NO (Norris and Ernst, 2018), the metabolism of LN resident cDCs may be affected by NO produced by Mtb-infected moDCs, resulting in effective cDC maturation for T cell activation.

Although many immunometabolic studies have been based on TLR agonist stimulation, Mtb can induce an Mtb-specific metabolic profile because it has components that induce various immune evasion mechanisms. Both Mtb lysate and LPS stimulation induced glycolysis in macrophages, but it was confirmed that Mtb infection significantly affected the metabolites of infected cells rather than simply increasing glycolysis, which showed a marked difference compared to LPS stimulation (Vrieling et al., 2020). These reports suggest that metabolic changes by Mtb infection or Mtb components should be studied not only in macrophages but also in various cells, including DCs. However, few studies have been conducted on the metabolic profile of DCs related to Mtb infection or its components. Guak et al. reported that glycolytic metabolism is essential for CCR7 oligomerization and DC migration (Guak et al., 2018). However, moDCs recruited to the infection site after Mtb infection exhibit a low CCR7 expression level, and migration to LNs does not occur effectively compared to that of non-infected moDCs (Harding et al., 2015). In addition, it has been reported that DC tolerance is induced by drugs promoting OXPHOS, such as vitamin D and dexamethasone (Ferreira et al., 2009; Ferreira et al., 2012; Basit and De Vries, 2019). These reports indicate the need to study the metabolic profile in the context of the mechanisms of inhibition of DC maturation by Mtb infection or specific components. In particular, study of the metabolic reprogramming of DCs with various CLR ligands or Ags involved in the immune evasion mechanism of Mtb discussed above may enable a deeper understanding of the function of DCs in TB. Given the importance and functional diversity of DCs reviewed in this paper, the Mtb-induced metabolic profile of DCs could be an important topic to be studied for TB control.

## DCs in genetic susceptibility to TB in animal models and humans

There is a spectrum of susceptibility among patients with TB (Vilaplana et al., 2010). There may be various causes, but in terms of the host, the genetic diversity of individuals may be a cause of susceptibility to Mtb (Vannberg et al., 2008; Davila et al., 2008; Leu et al., 2017; Zheng et al., 2017; Cai et al., 2019).

The inbred mouse model has a closed genetic background for each strain. Each strain of inbred mouse showed different survivability to Mtb infection; thus, it could be a rational model to study susceptibility to Mtb infection (Chackerian and Behar, 2003). Previous studies showed the correlation of susceptibility to Mtb infection with DCs in inbred mouse models (Medina and North, 1998; Chackerian and Behar, 2003; Yan et al., 2006). Relatively Mtb-resistant C57BL/6 and BALB/c mice significantly increased CD103^+^cDC1 in the lungs after 4 weeks of Mtb infection, whereas highly susceptible DBA/2 mice showed fewer CD103^+^ cDC1 recruited into the lungs (Leepiyasakulchai et al., 2012). The correlation of CD103^+^cDC1 recruitment with susceptibility was observed in LNs at 3 and 9 weeks after infection, and a higher number of IFN-γ^+^ cells was maintained at a higher level in resistant C57BL/6 compared to DBA/2 mice (Leepiyasakulchai et al., 2012). This correlation between susceptibility and DCs was even observed between relatively resistant C57BL/6 and relatively susceptible BALB/c (Sërgio et al., 2015). In addition, C57BL/6 mice showed approximately four times higher CCL19 gene expression in the lungs compared to BALB/c mice, showing differences in susceptibility according to the migration of DCs to LNs (Sërgio et al., 2015). The regulatory T cell population was not maintained in susceptible DBA/2 mice, whereas it was in resistant C57BL/6 mice (Cardona et al., 2015). Among the susceptible C3H strains, more susceptible C3HeB/FeJ mice showed lower regulatory T cell induction than C3H/HeN mice (Cardona et al., 2015). This phenomenon could be due to the role of CD103^+^cDC1 in suppressing excessive inflammation. These results imply that the number of cells and differences in the DC function can affect susceptibility.

Blischak et al. identified differentially expressed 645 genes between the DCs derived from PBMCs isolated from susceptible individuals (recovered from active TB) and resistant individuals (tested positive for latent TB). In addition, the identified genes were enriched for nearby SNPs with low p-values in TB susceptibility GWAS, indicating an association between genetic polymorphism and TB susceptibility (Blischak et al., 2017). Urazova et al. analyzed the association between the secretion of the proinflammatory cytokines IL-12р70, IL-18, and IL-27 by myeloid DCs and the presence of polymorphisms in their corresponding genes in 334 TB-patient samples and found that reduced IL-18 and IL-27 secretion and the polymorphisms leading to the altered secretion of IL-12p70 were associated with Mtb dissemination (Urazova et al., 2019). TB susceptibility associated with DC migration has also been reported in human studies. A study of 7.6 million genetic variants in 5530 patients with pulmonary TB and 5607 healthy controls recruited in Russia confirmed an association between TB susceptibility and variants of ASAP1 gene that encodes the DC migration regulator (Curtis et al., 2015; Waltl, 2015; Chen et al., 2019). However, there was no correlation between *ASAP1* and TB susceptibility in a Chinese population (Hu et al., 2016). In addition, the association of the *CD209* promoter single-nucleotide polymorphism (SNP)-336A/G with susceptibility to dengue, HIV-1, and TB in a study in sub-Saharan Africa has been reported (Vannberg et al., 2008). The latest meta-analysis confirmed that SNP-871A/G is associated with susceptibility to TB in all populations, and SNP-336A/G is a risk factor only for patients with TB in the Asian population (Yi et al., 2015). These reports show that DCs are an important population associated with TB susceptibility but show inconsistent results depending on the population, suggesting that further studies are required.

## Current development of anti-TB vaccines and DC-based immunological interventions

Among the various strategies to control TB, an effective TB vaccine can be the most cost-effective. BCG is currently the only licensed vaccine for TB; it is traditionally administered to neonates but has insufficient protection for pulmonary TB from adolescence, indicating that the development of improved vaccines is imperative (Andersen and Doherty, 2005). Various approaches have been attempted to overcome these obstacles, including subunit, recombinant BCG, and live attenuated vaccines. Several vaccine candidates are currently in clinical trials, addressing the key correlations of cellular or humoral immune responses with TB protection (Nell et al., 2014; Penn-Nicholson et al., 2015; Nemes et al., 2018; Van Der Meeren et al., 2018). Vaccine candidates are designed to potentiate DC function against Mtb, enhancing adjuvant efficacy, DC recruitment to/proliferation at the inoculation site, enhancing Ag uptake by DCs or exploiting immunogenic Ags from Mtb (**Table 3**).

**Table 3:** Mechanisms of the targets of DC-based approaches in TB vaccine candidates.

Concept	Product	Types	Immunological features	Ref.
DCs targeted vaccine	Anti-Dec-205-Ag85B	Conjugated vaccine	- Induction of Ag-specific humoral and cellular responses	(Stylianou et al., 2011)
		Conjugated vaccine for BCG booster	- T cell proliferation and IFN-γ production - No significant protection against Mtb challenge	
	α-DEC-ESAT	Conjugated vaccine	- Increased ESAT-6-specific IFN-γ producing CD4^+^ T cells	(Silva-Sanchez et al., 2015)
		Conjugated vaccine for BCG booster	- Increased IFN-γ^+^ production by specific T cells in the lungs - Ag-specific early (14 dpi) T cell response (IFN-γ production and CTL activity) - Reduced bacterial burden in lung	
	αDC-SIGN : Ag85B αDC-SIGN:P25	Conjugated vaccine	- Increase in Ag-specific IFN-γ^+^IL-2^+^TNF-α^+^ polyfunctional CD4^+^ T cells	(Velasquez et al., 2018)
	LV-AEG/SVGmu	Ag85A-ESAT-6 fusion protein (Ag85A-E6) expressing Lentivirus vector	- Induced strong Th1 response producing IFN-γ and IL-2 - Significantly increased levels of Ag85A-E6 specific IgG	(Shakouri et al., 2016)
Vaccine with DCs inducing signal	AdGM-CSF-adjuvanted BCG	Adjuvanted BCG vaccine	- Enhanced the magnitude and longevity of anti-mycobacterial type 1 immunity in LNs and spleen - Improved immune protection against secondary mycobacterial challenge	(Wang et al., 2002)
	BCG : GM‐CSF	Recombinant BCG vaccine	- Expands and activates APC in the lung and LNs - Accelerated priming of Ag-specific CD4^+^ T cells in the LNs - Increased migration of activated CD4^+^ T cells into lung	(Nambiar et al., 2010)
	BCG : GM-CSF	Recombinant BCG vaccine	- Increased numbers of dendritic cells in the dLNs at 7 and 14 days postvaccination - Enhanced expression of costimulatory molecules on migratory dendritic cells in the dLNs - Increase in the frequency of anti-mycobacterial IFN-γ-secreting T cells - 10-fold increase in protection against disseminated Mtb infection	(Ryan et al., 2007)
	BCG : Flt3L	Recombinant BCG vaccine	- Early expansion of DCs in dLNs - Increased safety on immunization with immunodeficient mice	(Triccas et al., 2007)
	pFlt-85	DNA vaccine	- Increased Ag85B specific IFN-γ production - Decreased bacterial burden in lung	
DCs transfer vaccine	LDC-Ag85	Cell-derived vaccines	- Increased infiltration of macrophages and lymphocytes into granulomas and parenchymal tissues - Increased numbers of CD4^+^ and CD8^+^ IFN-γ secreting cells	(Gonzãlez-Juarrero et al., 2002)
	BMDCs loaded with Mtb sonicate Ags	Cell-derived vaccines	- Significant increase in IFN-γ-producing cells in lungs and LNs	(Rubakova et al., 2007)
	DCs pulsed with Ag85A peptides	Cell-derived booster vaccines for MVA85A	- Immunized with DCs pulsed with both CD4^+^- CD8^+^-restrict epitopes together showed significant protection, but not with single peptide	(Mcshane et al., 2002)
	Ag85B-Z-DC	BCG booster vaccine	- Promote influx of CD4^+^ T cell into lung	(Griffiths et al., 2016)
		Booster vaccine for mucosal vaccine with Ag85B_240-254_ peptide	- Promote formation of B cell follicle formation in lung - Decreased bacterial burden in lung	
	AdAg85/DC (I.V.)	Mtb-Ag85A producing DCs	- Elicited a remarkably higher level of *ex vivo* IFN-γ production by CD4 and CD8 T cells at weeks 2, 6, and 12 post-immunization -Sustained levels of CD8 and CD4 CTL activity up to 12 weeks post-immunization.	(Malowany et al., 2006)
	AdAg85/DC (I.M.)	Mtb-Ag85A producing DCs	- Higher immunization efficacy than AdAg85/DC	

dpi, days post-infection; CTL, Cytotoxic T lymphocytes; BMDCs, bone marrow-derived dendritic cells; LNs, lymph nodes; BCG, Bacille Calmette-Guerin; dLNs, draining lymph nodes; I.V., intra-venous; I.M., intra-muscular.

Adjuvanted subunit vaccines provide effective protection by selecting proper Ags and adjuvants. The characteristics of adjuvants can affect the DC immune response causing effective T cell responses; therefore, various adjuvants have been developed and used in clinical trials. H4:IC31 (Bekker et al., 2020), H56:IC31 (Jenum et al., 2021), M72/AS01E (Tait et al., 2019), and ID93/GLA-SE (Coler et al., 2018) induced Ag-specific CD4^+^ T cells producing TNF-α, IFN-γ, and IL-2 simultaneously; several showed high levels of Ag-specific IgG (Coler et al., 2018; Tait et al., 2019). Immunization of Ag85B-ESAT-6 fusion protein with IC31 adjuvant can promote CD4^+^ T cell priming by inducing MHC class II activation and upregulation of costimulatory molecules on DCs (Kamath et al., 2008). AS01 is a liposome-based adjuvant of the M72/AS01E vaccine that contains two immunostimulants, monophosphoryl lipid A (MPL) and QS-21 (Garéon and Van Mechelen, 2011). AS01 induces DC activation, through the activation of NF-ĸB signaling by MPL (Casella and Mitchell, 2008) and Ag cross presentation by QS 21 (Ragupathi et al., 2011), resulting in enhanced adaptive immunity (Didierlaurent et al., 2014). GLA-SE, a synthetic TLR4 agonist formulated in a stable nano-emulsion of squalene oil, and mouse and human DCs stimulated with GLA produce IL-12 in a MyD88-and TRIF-dependent manner (Coler et al., 2011; Orr et al., 2013a); this adjuvant system induced a strong Th1 response to vaccine Ags (Coler et al., 2010; Orr et al., 2013a; Orr et al., 2013b; Orr et al., 2014). Subunit vaccines eventually deliver Ags *via* APC; thus, efficacy may vary depending on the formulation of the adjuvant used in the vaccination (Baldwin et al., 2012; Orr et al., 2013b)

Mtb-Ags capable of inducing DC maturation have been reported as potential TB vaccine targets. Mtb-Ags, such as RpfE, Rv0577, and MTBK_20640, induce DC maturation followed by IFN-γ producing Th1 and Th17 responses (Byun et al., 2012; Choi et al., 2015; Kwon et al., 2019). Rv0577 could induce the maturation of mouse splenic DCs *in vitro*, and these DCs could increase IFN-γ producing CD4^+^ and CD8^+^ T cells (Byun et al., 2012). MTBK_20640 induces DC maturation and Th1 response *in vitro* and vaccination with MTBK_20640 showed Ag-specific CD4^+^- CD8^+^- T cell responses with decreased bacterial burden and lung inflammation against the virulent Mtb HN878 strain (Kwon et al., 2019). Mtb Ag ESAT-6 fused with HSP90 (HSP90-E6) could mature DCs that induced Th1 and Th17 cell proliferation *in vitro* (Choi et al., 2020).

Whole-cell vaccines such as VPM1002 (Grode et al., 2013), MTBVAC (Tameris et al., 2019), RUTI (Vilaplana et al., 2010; Nell et al., 2014), DAR-901 (Lahey et al., 2010; Von Reyn et al., 2010), and MIP (Gupta et al., 2012; Mayosi et al., 2014) could induce an effective cellular immune response. BCG has advantages as a TB vaccine in terms of safety and universality; thus, studies on the usage of BCG for improved efficacy, such as BCG revaccination, recombinant BCG, and BCG-boosting vaccines are being conducted (Hoft et al., 2008; Orme, 2013; Kaufmann et al., 2014; Sander et al., 2015; Grøschel et al., 2017; Nemes et al., 2018). VPM1002 is a recombinant BCG that secrete listeriolysin (Hly) that increases *Listeria monocytogenes* phagosome escape (Grode et al., 2013) and promotes Hly activity by deleting ureC that inhibits phagosome lysosome fusion (Netea et al., 2006; Singh et al., 2010). This vaccine induces profound apoptosis in mouse and human APCs, allowing DCs to efficiently present Ags through the uptake of apoptotic vesicles (Grode et al., 2005). MTBVAC is a live, rationally attenuated Mtb with a deletion mutation in the virulence genes *phoP* and *fadD26*. Mutation of these genes impairs the synthesis of phthiocerol dimycocerosates (DIM) and trehalose-derived lipids, such as diacyl- (DAT) and polyacyl-trehaloses (PATs), which have DC immunomodulatory effects (Camacho et al., 1999; Cox et al., 1999; Gonzalo Asensio et al., 2006; Walters et al., 2006; Frigui et al., 2008; Gonzalo-Asensio et al., 2008). VPM1002 and MTBVAC induce not only higher Th1 activity but also Th17 activity in CD4 T cells than BCG (Nieuwenhuizen et al., 2017; Gupta et al., 2019; Dijkman et al., 2021), which may be due to the increase in the Ag-presenting ability of DCs through the mechanism described above or escape from the immunomodulatory effect. These reports suggest attempts to enhance vaccine efficacy by limiting the factors that inhibit DC function or enhancing the factors that aid in immunization, suggesting that further research on the role of DCs in TB pathogenesis is needed.

Owing to the above-mentioned DC characteristics, fundamental studies to produce effective TB vaccines based on the frequencies and functions of various types of DCs have been suggested (**Table 3**). After Mtb infection, DCs migrate to the dLNs and initiate primary protective Th1 responses. DCs are the only cells capable of priming naïve T cells (Bhatt et al., 2004). Thus, the limited number of DCs can affect the formation of protective immunity against Mtb infection. In this context, studies have been conducted to improve vaccine efficacy by increasing the number of DCs using growth factors such as GM-CSF or FMS-like tyrosine kinase 3 ligand (Flt3L). DNA vaccines encoding Mtb-Ags fused with GM-CSF or Flt3L showed improved protection against Mtb infection. For example, the Flt3L-Mtb32 DNA vaccine showed better protection against Mtb challenge in both the spleen and lungs than the BCG vaccine (Ahn et al., 2012). In another example, immunization using a DNA vaccine encoding mouse Mtb Ag85B with Flt3L elicited better protection than the DNA vaccine encoding Mtb Ag85B alone (Triccas et al., 2007). In addition, to increase the efficacy of BCG, an adenoviral GM-CSF transgene-based adjuvant formulation was used with BCG vaccination (Wang et al., 2002), resulting in markedly enhanced BCG immunogenicity and additional protection against Mtb infection with more APCs. Immunization of mice with BCG-encoded GM-CSF (Ryan et al., 2007; Nambiar et al., 2010) or Flt3L (Triccas et al., 2007) increased DCs in the lungs and mLNs, increasing BCG-reactive IFN-γ-secreting T cells with significant protection compared to immunization with BCG. These reports suggested that increasing DCs could be an important target for developing improved TB vaccines.

In addition to effective vaccine target Ags, the effective delivery of the Ags to DCs is an important aspect of an effective vaccine strategy. Dec-205 is an endocytic receptor (Inaba et al., 1995; Jiang et al., 1995; Mahnke et al., 2000) associated with Ag processing and presentation (Wang et al., 2000; Dudziak et al., 2007), and Mtb recognition (Von Garnier and Nicod, 2009). Furthermore, lung DCs in pulmonary TB express Dec-205 (Garcïa-Romo et al., 2004), indicating that Dec-205 could be a prominent target to deliver mycobacterial Ags. ESAT-6 conjugated with Abs targeting Dec-205^+^ DCs (α-DEC-ESAT) showed an ESAT-6-specific IFN-γ producing CD4^+^ T cell and reduced bacterial burden in a mouse model (Silva-Sanchez et al., 2015). Ag85B conjugated with Abs targeting Dec-205^+^ DCs (anti-Dec-205-Ag85B) induced Ag-specific cellular and humoral immune response, but no significant protection against Mtb infection (Stylianou et al., 2011). Similarly, vaccination with anti-DC-SIGN antibodies conjugated to Ag85B or peptide 25 of Ag85B targeting DC-SIGN^+^ DCs induces strong Ag-specific CD4^+^ T-cell response, but no protection against Mtb infection was observed (Velasquez et al., 2018). Although vaccine efficacy was not presented against Mtb infection, the DC-targeted vaccine using the lentivector LV-AEG/SVGmu encoding fusion protein Ag85A-ESAT-6 showed significant Th1 response and Ag-specific IgG. (Shakouri et al., 2016). Immunization *via* subcutaneous injection of BMDCs loaded with Mtb sonicate Ags showed increased survival and decreased bacterial burden against Mtb infection (Rubakova et al., 2007). Similarly, mouse intravenous immunization of DCs treated with Ag85A peptide showed a similar level of efficacy against Mtb infection as that of BCG (Mcshane et al., 2002). This protective effect of DC transfer can be accelerated by booster vaccination through the transfer of Ag85B-primed DCs following BCG vaccination (Griffiths et al., 2016). In addition, a genetically modified DC-based vaccine expressing Ag85A had increased vaccine efficacy compared with that of the DC vaccine administered following Ag85A treatment (Malowany et al., 2006). The number of DCs and their ability to form Th1/Th17 immunity are important factors in TB pathogenesis and defense against TB using vaccines.

The advantage of mRNA and adenovirus vector vaccine platform is that self-replicating viral vector vaccines or mRNA-based vaccines do not integrate into the intracellular nucleus, which is safe and can be effective even in small amounts, and compared to existing vaccine platforms, they can be produced quickly even in a small facility; therefore, it was possible to significantly reduce the time required to develop a vaccine for a disease, such as the COVID-19 pandemic (Corbett et al., 2020; Laczkõ et al., 2020; Mulligan et al., 2020; Mendonéa et al., 2021; Jacob-Dolan and Barouch, 2022). These advantages may enable efficient screening for the discovery of effective targets for TB vaccines. Xue et al. reported that the mRNA vaccine expressing Mtb-Ag MPT83 showed modest but significant protection against Mtb infection in a mouse model (Xue et al., 2004). But there is still no mRNA-based TB vaccine candidate in TB vaccine pipeline. Adenoviral vector TB vaccine has relatively more trials compared to mRNA-based TB vaccine, and vaccine candidates such as Ad5Ag85A and ChAdOx1.85A are currently in phase 1 clinical trials (Sable et al., 2019). Adenoviral vector- or mRNA-based vaccines can be generated by DC-targeting Abs fused with Mtb-Ag; hence, Mtb-Ag conjugated with DC-targeting Abs is capable of targeting DCs. In addition, it is possible to increase vaccine efficiency by promoting DC differentiation using growth factors-based vectors that promote DC differentiation. *Ex vivo* delivery of DCs could be a way to improve the stability of mRNA-based vaccines. For example, mRNA was transfected into DCs differentiated from the blood of a patient by electroporation, and then applied to a patient and used as a cancer vaccine (Gu et al., 2020; Wang et al., 2021). This method can be applied to the strategy of adoptive transfer of DCs for a TB vaccine. After transducing DC with a vector containing Mtb-Ag, adoptive transfer to a subject can increase the vaccine efficacy of the mRNA vaccine, since it enables continuous Mtb-Ag production *in vivo*. Rapid discovery of TB vaccine targets with the advantages of these new platforms will enable the combination of strategies with various targets and speed up the development of TB vaccines.

## Discussion

Currently, TB remains a globally uncontrollable disease, although Mtb was discovered as the causative agent of TB by Robert Koch in 1882. Several studies have been conducted, and valuable attempts have been made in various contexts, such as the independent and interdependent properties and functions of DC subsets in TB pathogenesis, their interactions with other immune cells, and immune evasion mechanisms through which Mtb avoids detection through DCs. One of the significant findings has been that DCs can induce immunity against TB while simultaneously being controlled by Mtb, suggesting that DCs are an important target for regulating the environment favored by the host or pathogen. Despite these advances, there are many conflicting opinions regarding the functions of the various DC subsets in Mtb infection, Mtb host-protective response-evasion mechanisms, and the crosstalk between DCs and other cells. Promising new vaccine candidates showing encouraging results are already in the pipeline, and ongoing studies have provided evidence to suggest that disease outcomes can be improved by understanding biological and cellular DC features for developing vaccine strategies.

**Figure 3:**
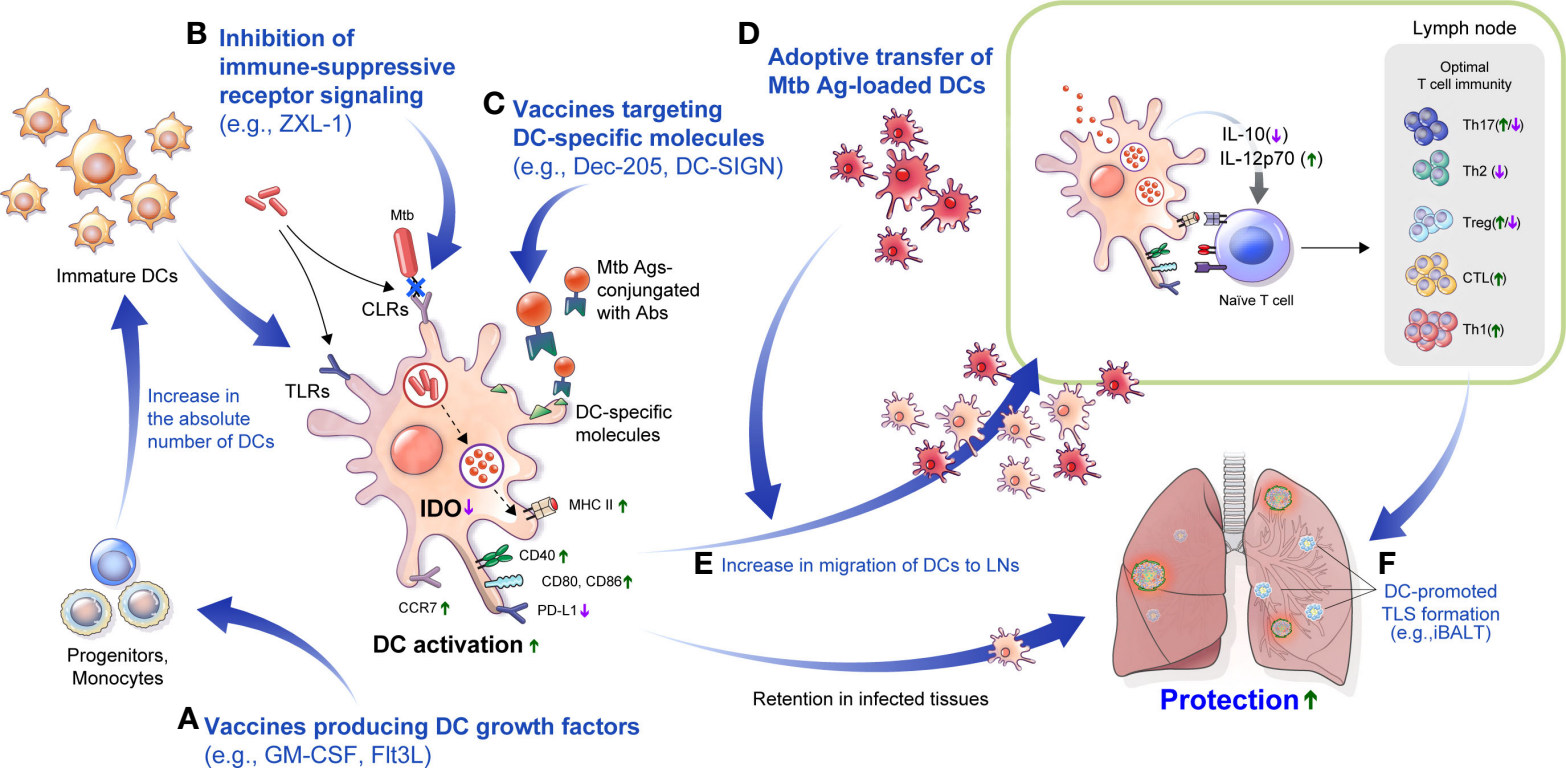
DC-based approaches to overcome TB disease. **(A)** Injection of Flt3L, GM-CSF or immunization with a vaccine that produces Flt3L, GM-CSF could increase the absolute number of DCs. **(B)** In Mtb infection, various CLRs that modulate the function of DCs could be blocked to promote DC maturation. For example, an aptamer such as ZXL-1 can inhibit the binding of ManLAM and mannose receptor. **(C)** Molecules such as DC-SIGN and Dec-205, mainly expressed on DCs, can be major targets of DC-targeted vaccines, which can be used to enable effective Ag delivery. **(D)** Adoptive transfer of DCs maturated with an Mtb-Ags increases the absolute number of DCs for interaction with T cells and can be used as a prime or booster vaccination, or as adjunctive therapy for antibiotic therapy to increase treatment efficiency. **(E)** Efficiently maturated DCs can interact with T cells through improved migration, which can help to configure optimal T cell immunity. **(F)** Efficient immunization with DCs can induce tertiary lymphoid structures formation such as iBALT, and can provide effective protection against subsequent infection. LNs, lymph nodes; Flt3L, FMS-like tyrosine kinase 3 ligand; GM-CSF, granulocyte-macrophage colony-stimulating factor; CLRs, C-type lectin receptors; DC-SIGN, DC-specific intercellular adhesion molecule-3 grabbing nonintegrin; TLS, tertiary lymphoid structure; iBALT, inducible bronchus–associated lymphoid tissue.

Furthermore, the features of DCs reviewed herein indicate that they could be a good target to potentiate host defense against TB (**Figure 3**). DC-targeted vaccines that can enable efficient protective immunity formation and an increase in the absolute number of DCs through the production of GM-CSF or Flt3L can also promote protective immunity (**Figure 3A**). The selective removal of Mtb components hindering DC function could help develop more effective TB control strategies (**Figure 3B**). For example, ZXL1 inhibits ManLAM-induced immunosuppression of Mtb-infected DCs by inhibiting the binding of Mtb ManLAM to MR (Pan et al., 2014). In addition, ZXL1 injection reduced the bacterial burden in mice and rhesus macaque models (Pan et al., 2014). Further, the effective delivery of Ags to DCs can be another strategy to control TB. As reviewed above, by conjugating Mtb-Ags to an Ab, such as Dec-205, that targets a DC-specific molecule, efficient DC antigen delivery, and immune response can be induced (**Figure 3C**). In addition to promoting DC differentiation, there is a method for the adoptive transfer of DCs induced by maturation with Mtb-Ags (Griffiths et al., 2016) to form protective immunity by increasing DCs in the host (**Figure 3D**). It is possible to induce effective DC maturation for Mtb protective immunity using carefully selected Mtb-Ags rather than DCs with reduced function due to Mtb infection that could be used as a prime or booster vaccine or adjunctive immunotherapy to increase the effectiveness of antibiotic regimens.

Besides there are strategies to inhibit Mtb-induced immunosuppressive factors in the host. One of the most common features of the Mtb immune-evasion mechanism *via* DCs is the increased secretion of IL-10 to induce tolerogenic DC generation *in vitro* (Mcbride et al., 2002; Torres-Aguilar et al., 2010). IL-10 secretion in Mtb-infected DCs is correlated with the virulence of Mtb strains, and the maturation phenotype was recovered by blocking IL-10 signaling, enhancing T cell response with reduced bacterial burden in mouse models (Beamer et al., 2008; Kim et al., 2017). Furthermore, IDO inhibition using 1-methyl-tryptophan increased Mtb killing, increased lymphoid follicles and pulmonary lymphocyte proliferation, and improved disease outcome (Gautam et al., 2018). These methods can increase the migration of DCs to LNs and enable effective interaction with T cells (**Figure 3E**). In addition, there is a correlation between iBALT formation and protection in Mtb infection, and DCs play an important role in the formation and maintenance of this structure. Therefore, these DC targeted strategies could promote the formation of iBALTs, which plays a protective role against Mtb infection (**Figure 3F**). Comprehensive regulation and application of the DCs properties in Mtb infection can be used to develop vaccines or treatment strategies for TB control.

GM-CSF can play a pathogenic role in autoimmune diseases such as multiple sclerosis and rheumatoid arthritis (Lotfi et al., 2019). Flt3L also has the potential to play a pathogenic role in autoimmune thyroid disease or rheumatoid arthritis (Dehlin et al., 2008; Wilson et al., 2021). Currently, vaccines using GM-CSF or Flt3L as adjuvants have been demonstrated to be safe and effective in cancer vaccine clinical studies, but in study with gynecological cancer patients, some patients receiving FLt3L or FLT3L plus GM-CSF as an adjuvant showed autoimmune side effects, such as rash and Sica syndrome (Disis et al., 2002). Therefore, for safety, these potential risks must be considered when developing vaccines encoding endogenous molecules such as Flt3L or GM-CSF. Vaccination *via* DC adoptive transfer showed protective efficacy against Mtb infection close to that of BCG and significant protection when used as a booster vaccine for BCG vaccine. Although these reports indicate that DCs can be effective targets for TB vaccines, their use as a practical vaccine has limitations. For direct DC adoptive transfer, isolation of CD14^+^ monocytes through apheresis is required, and infrastructure for DC differentiation and cytokines for cell culture are required (Yu et al., 2022). In addition, intranasal Ag85A-primed DC transfer induced excessive inflammation in lung tissue although it could induce IFN-γ producing T cells (Gonzãlez-Juarrero et al., 2002). Considering that TB mainly affects people in resource-constrained settings, primed DC transfer would be difficult to implement. In such contexts, DC-like biomimetic nanoparticles used in immunotherapy for breast cancer (Li et al., 2021) could be the potential alternatives in patients who have difficulty using autologous DCs. DMSNs^3^@HA is a DC-like nanoparticle modified with hyaluronic acid to target CD44 overexpressed on cancer cells, and conjugated with anti-CD3, anti-CD28 to interact with T cell, and anti-PD-1 to block the PD-1/PD-L1 pathway. DMSNs^3^@HA synergistically activates T cells and improves their immune response to significantly inhibit the progression of breast cancer (Li et al., 2021). This DC-like nanoparticle can be an alternative to adoptive transfer of DCs for TB vaccination through conjugation with Abs loaded with proper Ags. Since it enables targeting specific cell populations, effective TB control can be achieved by regulating the cell-to-cell interactions described above through immunological interventions, such as cell-specific drug delivery or the induction of specific types of apoptosis. Therefore, DC-targeted tactics such as nanoparticles or Abs (e.g., anti-DC-SIGN Ab or anti-Dec-205 Ab) can be used in anti-TB vaccines or immunotherapies.

Despite their importance, there are relatively few reports on the immune responses to antibiotic treatment. Effective therapeutic vaccines can shorten treatment and disease severity and increase protective immunity (Larsen et al., 2018; Chuang et al., 2020). DCs could be an alternative therapy to increase protective immunity during antibiotic therapy. Elucidating the principles of an effective immune response could help develop a more effective anti-TB vaccine and increase the effectiveness of TB treatment. Exploiting immune interventions using or targeting DC properties regulated by interactions with other cellular compartments, Mtb, or its Ags in a disease stage-specific manner could be a novel way to improve TB control effectively.

The complexity of immune response to different disease stages and local immune environments makes it difficult to identify the ideal immune response that is equivalent to that induced by a vaccine or immune therapy to control TB. For example, the Th17 response, which plays a protective role in the early stage of infection, may induce excessive neutrophilic inflammation, leading to tissue damage in the chronic stage. Anti-PD-1 Ab treatment suppresses excessive inflammation but can induce progression to active TB in patients with latent TB (Tezera et al., 2020). TNF-α is a proinflammatory cytokine activating immune cells to inhibit Mtb growth. The inhibition of PD-1 signaling increases the production of TNF-α, which leads to accelerated bacterial growth (Kauffman et al., 2021). We reviewed the dual role of DCs in protection and pathogenicity during Mtb infection, and found that understanding and regulating the duality of DCs could be a starting point for achieving the ideal immune response to control TB.

## Author contributions

All authors listed have made a substantial, direct and intellectual contribution to the work, and approved it for publication.

## Funding

This review was supported by the Korea Health Technology R&D Project through the Korea Health Industry Development Institute (KHIDI), funded by the Ministry of Health and Welfare, Republic of Korea (HV20C0144), and a National Research Foundation of Korea (NRF) grant funded by the Korean government (MSIT) (NRF-2021R1I1A1A01052391), Republic of Korea. The funders had no role in the decision to publish or prepare this manuscript.

## Acknowledgments

The authors thank Medical Illustration & Design (MID), a part of the Medical Research Support Services of Yonsei University College of Medicine, for all artistic support related to this work.

## Conflict of Interest

The authors declare that this review was conducted without any commercial or financial relationships that could be construed as a potential conflict of interest.

## Publisher’s note

All claims expressed in this article are solely those of the authors and do not necessarily represent those of their affiliated organizations, or those of the publisher, the editors and the reviewers. Any product that may be evaluated in this article, or claim that may be made by its manufacturer, is not guaranteed or endorsed by the publisher.
